# De novo transcriptome revealed genes involved in anthocyanin biosynthesis, transport, and regulation in a mutant of *Acer pseudosieboldianum*

**DOI:** 10.1186/s12864-022-08815-y

**Published:** 2022-08-08

**Authors:** Jia-Lin Li, Zhuo Weng, Xin-Yu Li, Bo Xu, Yu-Fu Gao, Li-Ping Rong

**Affiliations:** College of Agriculture, Yanbian University, Yanji, 133002 China

**Keywords:** *Acer pseudosieboldianu*, Transcription, Mutant, Anthocyanin, Candidate genes

## Abstract

**Background:**

*Acer pseudosieboldianum* is a kind of excellent color-leafed plants, and well known for its red leaves in autumn. At the same time, *A. pseudosieboldianum* is one of the native tree species in the northeast of China, and it plays an important role in improving the lack of color-leafed plants in the north. In previous study, we found a mutant of the *A. pseudosieboldianum* that leaves intersect red and green in spring and summer. However, it is unclear which genes cause the color change of mutant leaves.

**Results:**

In order to study the molecular mechanism of leaf color formation, we analyzed the leaves of the mutant group and the control group from *A. pseudosieboldianum* by RNA deep sequencing in this study. Using an Illumina sequencing platform, we obtained approximately 276,071,634 clean reads. After the sequences were filtered and assembled, the transcriptome data generated a total of 70,014 transcripts and 54,776 unigenes, of which 34,486 (62.96%) were successfully annotated in seven public databases. There were 8,609 significant DEGs identified between the control and mutant groups, including 4,897 upregulated and 3,712 downregulated genes. We identified 13 genes of DEGs for leaf color synthesis that was involved in the flavonoid pathway, 26 genes that encoded transcription factors, and eight genes associated with flavonoid transport.

**Conclusion:**

Our results provided comprehensive gene expression information about *A. pseudosieboldianum* transcriptome, and directed the further study of accumulation of anthocyanin in *A. pseudosieboldianum*, aiming to provide insights into leaf coloring of it through transcriptome sequencing and analysis.

**Supplementary Information:**

The online version contains supplementary material available at 10.1186/s12864-022-08815-y.

## Background

As living standards rise, the use of monotonous colors in urban greening has been insufficient to meet landscape development needs. To provide relief from color monotony, plants with brightly colored leaves and aesthetic characteristics are highly desirable. Such plants are widely used in landscaping, and color is used to attract the attention of people to the cultivation of new plant varieties. Plants with colored leaves are plants with non-green leaves that have colors other than green in all or part of their leaves throughout the growing season or at a certain stage thereof [1]. The pigment content of leaves is the key factor that affects colored leaves in plants. Accordingly, chlorophyll, carotenoid, and anthocyanin are the three most important pigments affecting color in leaves. Flavonoid anthocyanins are the determining pigments for the formation of red leaves, and anthocyanin plays a leading role in the formation of red leaves. The colors of the plant organs are mainly attributed to the accumulation of anthocyanins, a class of plant flavonoid metabolites [2–4]. The anthocyanin biosynthetic pathway has been well studied in plants. Anthocyanin biosynthetic pathway is a branch of phenylalanine biosynthetic pathway [3]. The structural genes in anthocyanin synthesis pathway can be divided into upstream and downstream gene groups. In fact, the synthesis of anthocyanins shares the same upstream pathways with proanthocyanidins and flavonol derivatives [5]. Phenylalanine is the precursor of flavonoid, which is used as substrate, phenylalanine ammonia lyase (PAL), cinnamate-4-hydroxylase (C4H) and 4-coumaroyl-CoA synthase (4CL) catalyze a series of reactions to produce 4-coumaroyl-CoA. The catalysis of chalcone synthase (CHS) can catalyze the synthesis of chalcones. Subsequently, after the action of chalcone isomerase (CHI), the basic three rings of the general C6-C3-C6 flavonoid skeleton is produced. The B ring of the naringenin flavanone can be further hydroxylated by flavonoid-3’-hydroxylase(F3’H) or flavonoid-3′5’-hydroxylase (F3′5’H) to form eriodictyol or pentahydroxyflavanone. The naringenin, eriodictyol and pentahydroxyflavanone can be modified by the catalysis of flavanone-3β-hydroxylase (F3H) to form the corresponding dihydrokaempferol, dihydroquercetin and dihydromyricetin, respectively. Besides, the dihydrokaempferol can also be catalyzed by F3’H or F3′5’H to produce other two dihydroflavonols, dihydroquercetin or dihydromyricetin [6]. In the downstream pathways, dihydroflavonol-4-reductase (DFR) catalyzes these dihydroflavonols to form their corresponding leucoanthocyanidins. And the anthocyanidins were catalyzed by anthocyanidin reductase (ANR) to produce the substrates for the proanthocyanidins synthesis. Finally, anthocyanins with different colors were produced by the joint action of glycosyl transferases (GT), methyl transferase (MT) and acyl transferase (AT) [7]. The enhanced expression of structural anthocyanin biosynthesis genes directly accounts for increased levels of anthocyanin accumulation in plants [8]. These structural genes are regulated mainly at the transcriptional level [9]. In the anthocyanin biosynthetic pathway, some regulated genes encode transcription factors (TFs) that combine the promoters of structural genes to regulate their expression levels. Some of the R2R3-MYB, bHLH (basic helix-loop-helix), and WD40 TFs are significantly related to anthocyanin biosynthesis and are involved in the formation of a MYB-bHLH or MYB-bHLH-WD40 (MBW) complex [10, 11]. The combinations and interactions between these TFs play an important role in regulating the anthocyanin biosynthetic pathway. At present, some progress has been made in the investigation of the role of anthocyanin in red leaves. Luo et al. [12] found that anthocyanins caused the red color in leaves. Furthermore, Jiang et al. [13] analyzed the relationship between the change in leaf color and the genes that affect anthocyanin accumulation, and Huang [14] showed that red leaves contained a large amount of anthocyanin by studying the physicochemical properties of *Acer palmatum* leaves.

Maples (*Acer* genus), with red or yellow autumn leaves, are plants well-known for their colored leaves. The bright color of maple leaves is caused by a large amount of anthocyanin in the leaves [15]. In recent years, researchers have attached great importance to the molecular study of maple leaf color and made great achievements in this area. Cai et al. [16] found that the interaction between cyanidin 3-galactoside content and chlorophyll content was an important factor in leaf color change from red to green in *A. palmatum.* Guo [17] found seven anthocyanin-related TFs in *Acer rubrum* leaves during leaf color transformation that were mainly mediated by mechanisms including propane biosynthesis, anthocyanin biosynthesis, and flavonoid biosynthesis. These studies lay a foundation for further research into the molecular genetic mechanisms of anthocyanin synthesis and accumulation in leaves, and the breeding of new varieties of plants with colored leaves.

*A. pseudosieboldianum* is a deciduous tree in family Aceraceae, and is one of the most highly sought-after ornamental trees in landscaping due to its fiery red leaf color and graceful leaf shape in autumn [18, 19]. It is native to eastern Russia, northern Korea, and China. In China, it is distributed throughout eastern and southeastern Heilongjiang, southeastern Jilin, and eastern Liaoning. It is a native tree with excellent landscaping application prospects in northeast China [20]. Its appearance is graceful and pleasant, and its leaves are red and colorful in autumn, therefore, it has high ornamental value and is an essential ornamental plant in landscaping.

The leaves of most maples are green in spring and summer and change to red in the autumn. However, we found a mutant of *A. pseudosieboldianum* with leaves that shifted between red and green in spring and summer. The objective of this study was to identify the genes causing the color change in maple leaves. Complete genome sequencing provides a great deal of genetic information, but sequencing is limited by high costs. RNA-seq analysis represents a cost-effective tool for the discovery of functional genes. To date, advances in next-generation sequencing technologies have enabled the simultaneous generation of an enormous amount of data [21]. In this study, the transcriptome was used to analyze the genes involved in the flavonoid pathway in *A. pseudosieboldianum.* The de novo transcriptome data generated in this study will allow for the elucidation of the mechanism of anthocyanin synthesis during mutation development and enrich plant databases and will eventually serve as reference sequences for other plant species.

## Results

### Anthocyanin content analysis

**Fig. 1:**
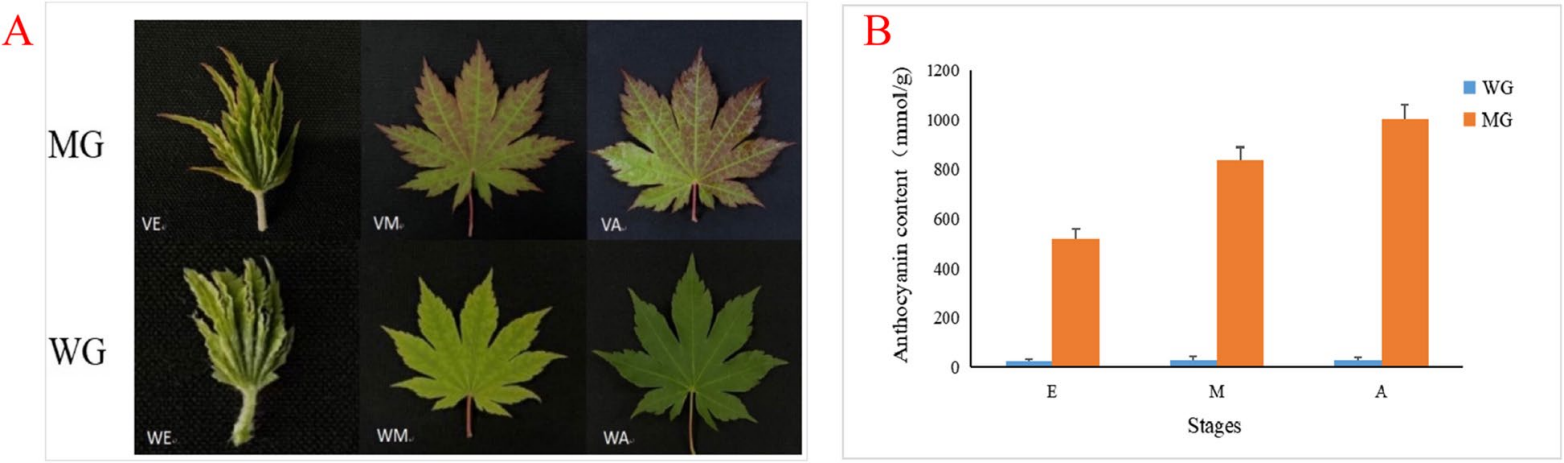
The morphology (**A**) and the anthocyanin content (**B**) of leaves in *A. pseudosieboldianum* during early, middle and late stages from wild and mutant groups

To clarify the differences underlying the leaf coloration between the wild group (WG) and its mutants (MG), phenotypes (Fig. 1A) and content anthocyanin (Fig. 1B) from three developmental stages, the early (E), middle (M) and late stages (A) were analyzed. As was shown in Fig. 1B that the anthocyanin content of MG increased gradually during the leaf color transformation and in contrast, the anthocyanin content of WG was little and the change was not obvious (Fig. 1B). The result indicated that anthocyanin is one of the important indexes that affect the leaf color of maple variation group.

### Sequencing and de novo assembly

To elucidate the molecular mechanism of leaf-color changes in *A. pseudosieboldianum*, 18 mRNA samples (WE, WM, WA and VE, VM, VA; in triplicate) were sequenced on the Illumina sequencing platform. In total, there were 390,877,630 raw reads were generated from the *A. pseudosieboldianum* transcriptome. After the low-quality reads were removed, 276,071,634 clean reads were obtained. The percentage of the Q30 base was 91.55% or above. After obtaining high-quality sequencing data, the sequences were assembled using Trinity, an assembly software designed for high-throughput transcriptome sequencing. This produced 70,014 transcripts with N50 lengths of 2,152 bp and 54,776 unigenes with N50 lengths of 2,073 bp. The unigenes had a minimum length of 190 bp, a maximum length of 17,342 bp, and a total length of 44,457,414 bp (Table 1).

**Table 1:** Statistics of transcriptome assembly and unigenes

	**Transcripts**	**Unigenes**
Number	70,014	54,776
Total length (bp)	67,202,571	44,457,414
Maximum length (bp)	17,342	17,342
Minimum length (bp)	190	190
Average length (bp)	959	811
N50 length (bp)	2152	2073

### Functional annotation and classification

In total, 34,486 unigenes, accounting for 62.96% of all the unigenes, were successfully annotated to the seven databases, including the COG, GO, KEGG, KOG, Pfam, Swissprot, and nr databases (Table 2). During GO annotation, 19,372 unigenes were divided into three functional categories, including cellular components (CC; 36,577 unigenes), biological processes (BP; 28,731 unigenes), and molecular functions (MF; 36,577 unigenes). These unigenes were clustered into 53 GO terms,respectively. Furthermore, CC and BP were clustered into 15 and 23 GO terms, respectively, with the ‘membrane’ (7,177 unigenes) and ‘metabolic process’ (9,500 unigenes) subcategories being the largest. In the MF category, these matched unigenes were annotated to 15 GO terms, with the two top terms being ‘catalytic activity’ (9,616 unigenes) and ‘binding’ (9,425 unigenes).

**Table 2:** All unigenes of the transcriptome annotated in the different public databases

Database	Number of unigenes
COG annotation	10,596
GO annotation	19,372
KEGG annotation	21,833
KOG annotation	17,590
Pfam annotation	19,401
Swiss-prot annotation	24,180
Nr annotation	32,814

KOG analysis revealed 17,590 unigenes that were divided into 25 groups. The largest group was R (general function prediction only) with 3778 unigenes (19.24%), followed by T (signal transduction mechanisms) with 2,142 unigenes (10.91%).

A total of 21,833 unigenes were assigned to 358 KEGG pathways. These KEGG pathways were clustered into five branches: carbon metabolism (803 unigenes), 2-oxocarboxylic acid metabolism (163 unigenes), fatty acid metabolism (163 unigenes), degradation of aromatic compounds (22 unigenes), and biosynthesis of amino acids (724 unigenes). These genes were analyzed for gene mining and provided a valuable resource for the functional analysis of *A. pseudosieboldianum.*

### Identification and functional analysis of differentially expressed genes (DEGs)

**Fig. 2:**
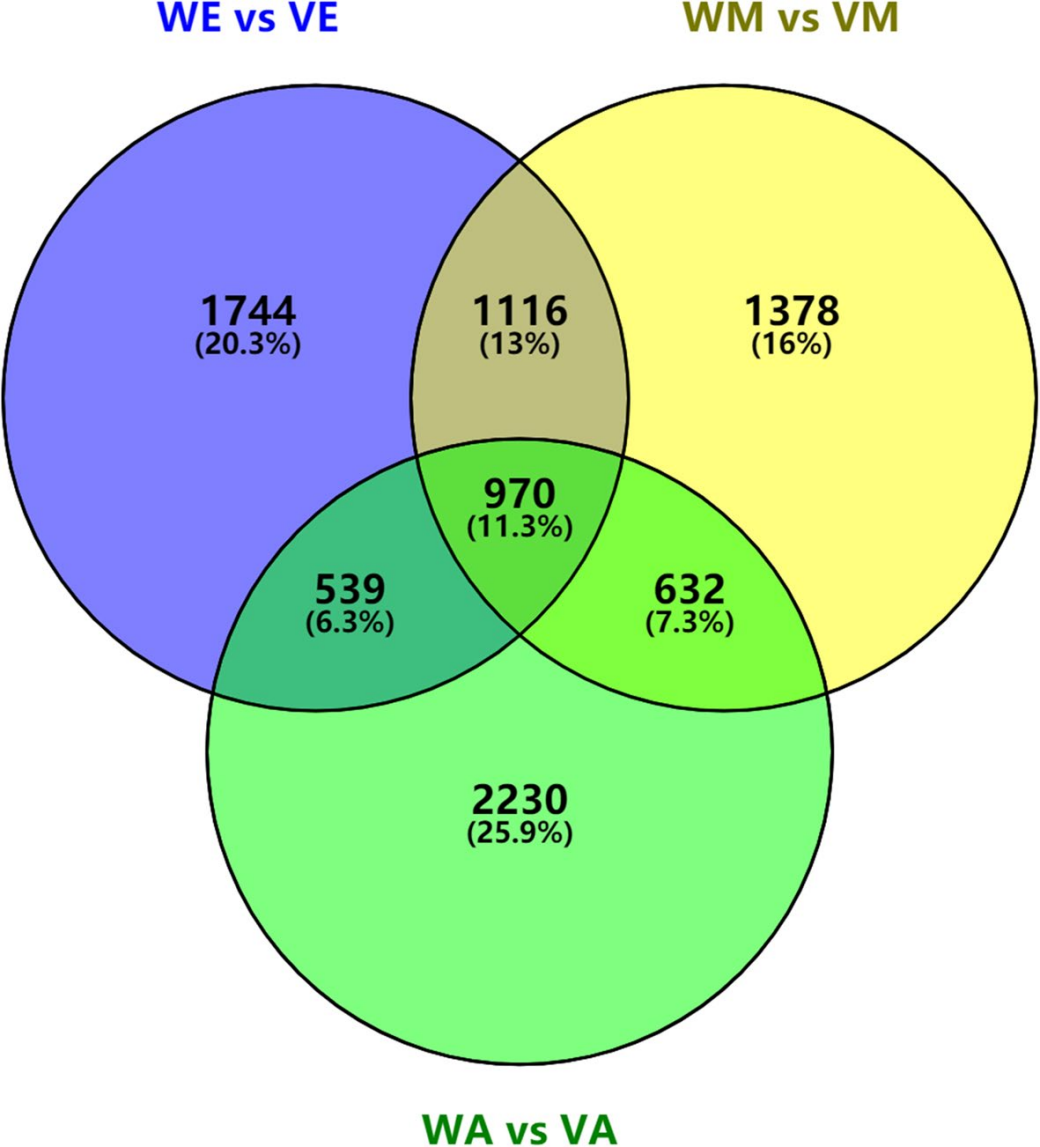
Venn Diagram of DEGs at various stages of *A. pseudosieboldianum* development between three stages. (WE vs VE、WM vs VM、WA vs VA)

To explore anthocyanin biosynthesis-related genes in *A. pseudosieboldianum* at different color transition stages, DEGs of *A. pseudosieboldianum* at different color transition stages were compared. A total of 8,609 significant DEGs, including 4,897 up-regulated and 3,712 down-regulated genes, were found in the control and mutant groups (Table 3). The Venn diagram of DEGs at various stages is shown in Fig. 2.

**Table 3:** Statistics of the number of DEGs in the control group and the mutant group during the three stages of leaf color change

	**All DEGs**	**up-regulated**	**down-regulated**
WE1_WE2_WE3_vs_VE1_VE2_VE3	4369	2514	1855
WM1_WM2_WM3_vs_VM1_VM2_VM3	4096	2168	1928
WA1_WA2_WA3_vs_VA1_VA2_VA3	4371	2071	2300
All DEGs Number	8609	4897	3712

**Fig. 3:**
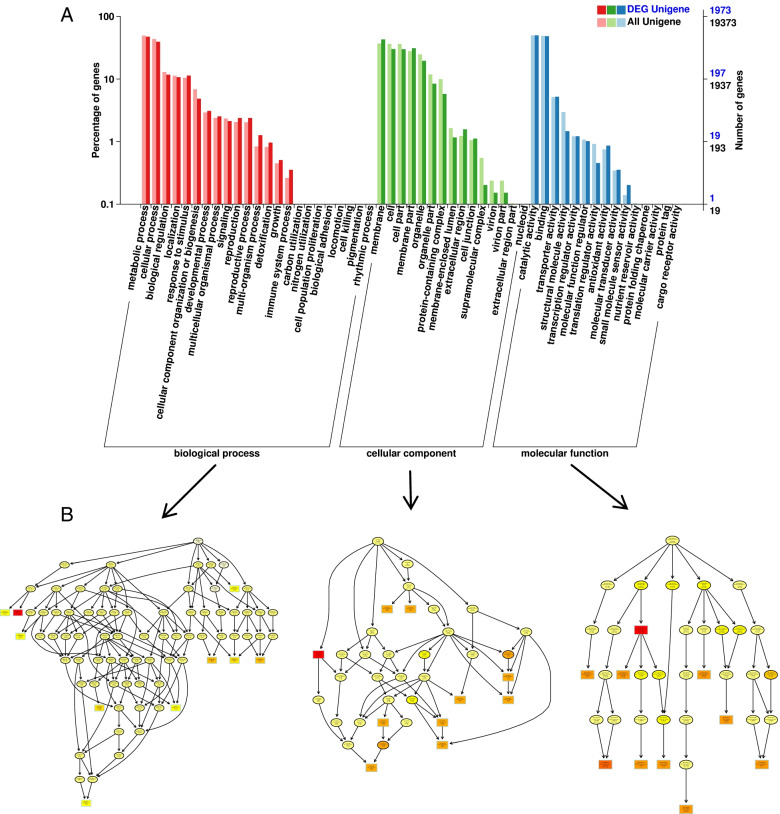
GO annotation of DEGs (WA vs VA) **A** GO enrichment histogram. **B** Thumbnail view of directed acyclicgraphs (DAGs) of BP, CC, and MF

To accurately identify and classify the functions of each DEG, classification and enrichment GO analyses were conducted in the control group and the mutant group in the early, middle, and late stages. The enrichment degrees of the metabolic process and cellular process categories were the highest in the BP category, and the results showed that the enrichment degree of the catalytic activity and the binding was highest in the MF category. In the CC category, the top GO terms were ‘membrane’, ‘cell’, and ‘membrane part’. The GO annotations of DEGs for WA vs VA are shown in Fig. 3. In addition, the GO annotation results of DEGs for WE vs VE and WM vs VM are shown in Fig. S1 and Fig. S2 respectively. All annotated unigenes were divided into three functional GO categories (Fig. 3A), consisting of the biological process (BP), the cellular component (CC), and the molecular function (MF) categories. The number of corresponding genes is also shown in the figure. Three GO DAGs (Fig. 3B) were constructed for the three GO domains (BP, CC, and MF). The top three enriched GO terms were enriched in the DAG of MF. The most significant enrichment (shown in red) was detected in the oxidoreductase activity of molecular functionality (GO: 0,016,491).

**Fig. 4:**
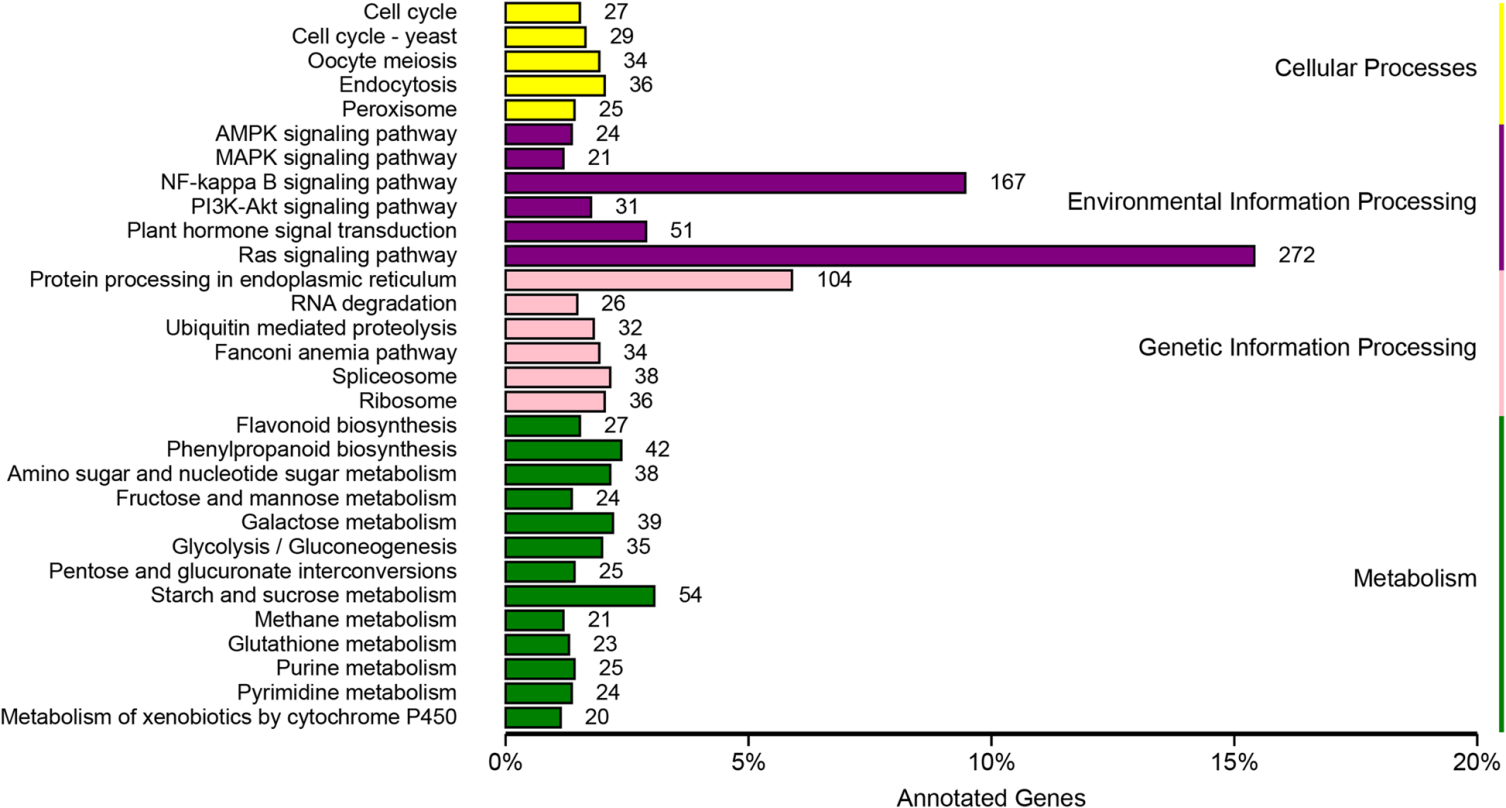
KEGG annotation of DEGs (WA vs VA)

KEGG analysis can help to better elucidate specific processes, gene functions, and gene interactions at the transcriptomic level. To identify the DEGs involved in leaf discoloration during development, a KEGG pathway enrichment analysis of DEGs in the leaves of *A. pseudosieboldianum* was conducted to further reveal the functions of DEGs. During the KEGG comparison, three comparisons were made between the control group and the mutant group in the early, middle, and late stages. The KEGG enrichment analysis of WA and VA (Fig. 4) revealed that the most significantly enriched pathway was the RAS signaling pathway, and the second most significantly enriched pathway was the NF-kappa B signaling pathway. The results were the same in the early and middle periods. The KEGG annotation of DEGs for WE vs VE and WM vs VM are listed in Fig. S3 and Fig. S4, respectively.

### Identification of candidate genes involved in the anthocyanin biosynthesis

This study identified 13 candidate unigenes encoding seven enzymes related to the flavonoid biosynthesis pathway (ko00941) in the *A. pseudosieboldianum* transcriptome. The 13 genes with significant differences in flavonoid biosynthesis are as follows: one CHS gene, two CHI genes, one F3H gene, three F3’H genes, one DFR gene, three ANS gene, and two ANR genes(Table 4).

**Table 4:** Statistics of candidate genes involved in the flavonoid biosynthesis

Gene name	ID	Stage	Regulated	Log2FC
CHS	TRINITY_DN452_c0_g1	WE vs VE	up	3.755269715
		WM vs VM	up	2.033196146
		WA vs VA	up	2.736729279
CHI	TRINITY_DN3806_c0_g1	WE vs VE	-	-
		WM vs VM	down	-1.014599085
		WA vs VA	-	-
	TRINITY_DN2599_c0_g1	WE vs VE	-	-
		WM vs VM	-	-
		WA vs VA	up	1.053398091
F3H	TRINITY_DN4505_c0_g1	WE vs VE	-	-
		WM vs VM	down	-1.687898465
		WA vs VA	up	2.906521555
F3'H	TRINITY_DN9177_c0_g1	WE vs VE	down	-2.149433931
		WM vs VM	-	-
		WA vs VA	-	-
	TRINITY_DN1592_c0_g1	WE vs VE	-	-
		WM vs VM	-	-
		WA vs VA	up	1.390888323
	TRINITY_DN10336_c0_g1	WE vs VE	-	-
		WM vs VM	-	-
		WA vs VA	down	-3.504240445
DFR	TRINITY_DN3960_c0_g1	WE vs VE	up	3.547581949
		WM vs VM	up	1.362813351
		WA vs VA	up	2.502763118
ANS	TRINITY_DN18024_c0_g1	WE vs VE	up	2.42177804
		WM vs VM	-	-
		WA vs VA	up	3.606238162
	TRINITY_DN21360_c0_g4	WE vs VE	down	-3.510028167
		WM vs VM	down	-3.84212978
		WA vs VA	down	-1.486794701
	TRINITY_DN4873_c0_g1	WE vs VE	down	-2.005736745
		WM vs VM	down	-1.732875953
		WA vs VA	down	-2.933684766
ANR	TRINITY_DN11089_c0_g3	WE vs VE	down	-2.063110906
		WM vs VM	-	-
		WA vs VA	-	-
	TRINITY_DN12211_c0_g1	WE vs VE	-	-
		WM vs VM	down	-2.883606996
		WA vs VA	-	-

### Identification of candidate genes involved in flavonoid transport

In this transcriptome, 119 DEGs were genes encoding transporters (Table 5), including ABC transporters (Tables S1), glutathione S-transferase (GST) (Tables S2), multidrug resistance-associated proteins (MRPs) (Tables S3), multidrug and toxic compound excretion-associated proteins (MATEs) (Tables S4), and H + -ATPases (Tables S5). These genes may be involved in the transport of flavonoids from cytoplasmic synthesis to vacuolar accumulation in *A. pseudosieboldianum*. This may be very important for the study of leaf color mutant genes in *A. pseudosieboldianum*. Among these genes, six GST genes, one MATE gene and one H + -ATPase gene were found to be related to the transport of flavonoids (Table 6).

**Table 5:** Statistics of different gene transporters of DEGs

Genes	Transporters number of DEGs
ABC transporters	54
GST	23
MRPs	13
Mate Family	14
H + -ATPases	15
All transporters	119

**Table 6:** The transporter genes identified in differentially expressed genes for leaf color synthesis

Gene name	ID	Log2FC	Regulated	KEGG annotation
GST	TRINITY_DN27953_c0_g1	2.681762358	up	K00799 hypothetical protein; glutathione S-transferase
	TRINITY_DN2666_c0_g1	3.975348379	up	K00799 glutathione S-transferase F11-like; glutathione S-transferase
	TRINITY_DN20109_c1_g2	4.32562752	up	K05022 glutathione S-transferase DHAR3, chloroplastic; K21888 glutathione dehydrogenase/transferase
	TRINITY_DN10065_c0_g1	1.075317256	up	K00799 hypothetical protein; glutathione S-transferase
	TRINITY_DN74582_c0_g1	1.16577486	up	K00799 glutathione S-transferase F6; glutathione S-transferase
	TRINITY_DN1196_c0_g2	-1.064734174	down	K00799 hypothetical protein; glutathione S-transferase
MATE	TRINITY_DN12742_c0_g1	2.059229886	up	K03327 protein DETOXIFICATION 34; multidrug resistance protein, MATE family
	TRINITY_DN5404_c1_g1	1.085133081	up	K01535 ATPase 11, plasma membrane-type; H + -transporting ATPase

### Identification of candidate transcription factors (TFs) involved in the anthocyanin biosynthetic

To investigate the regulatory mechanism of anthocyanin and flavonoid biosynthesis genes in *A. pseudosieboldianum*, the transcripts of TFs were analyzed. The differentially expressed TFs are divided into different families, among which MYB, bHLH, and WD40 TFs play extremely important roles in regulating anthocyanin and flavonoid accumulation. The results of the present study showed that 214, 112, and 16 genes were predicted to encode MYB, bHLH, and WD40 proteins, respectively, in the transcriptomic database. The DEGs are listed in Tables S6, S7, S8. Among them, 19 MYB genes and seven bHLH genes were identified to be related to leaf color synthesis according to the KEGG analysis (Table 7).

**Table 7:** The transcription factors identified in differentially expressed genes for leaf color synthesis

Gene name	ID	Log2FC	Regulated	KEGG annotation
MYB	TRINITY_DN13344_c0_g1	-2.901362299	down	K00512 flavonoid 3',5'-hydroxylase-like
	TRINITY_DN7218_c0_g1	-2.854520525	down	K09754 5-O-(4-coumaroyl)-D-quinate 3'-monooxygenase
	TRINITY_DN73935_c0_g1	1.901287881	up	K00512 flavonoid 3'-monooxygenase-like
	TRINITY_DN826_c0_g1	2.106435083	up	K09422 transcription factor MYB23; transcription factor MYB, plant
	TRINITY_DN8618_c0_g1	1.251017941	up	K09422 transcription factor MYB, plant
	TRINITY_DN2186_c1_g1	-1.802818464	down	K13264 isoflavone 7-O-glucoside-6''-O-malonyltransferase
	TRINITY_DN3207_c1_g1	-1.187986048	down	K13264 isoflavone 7-O-glucoside-6''-O-malonyltransferase
	TRINITY_DN1359_c1_g1	2.475553161	up	K09422 transcription factor MYB114 isoform X2; transcription factor MYB, plant
	TRINITY_DN2377_c1_g1	1.639912152	up	K09422 transcription factor MYB114; transcription factor MYB, plant
	TRINITY_DN82895_c0_g1	2.407399588	up	K0942 low quality protein: transcription factor MYB114-like; transcription factor MYB, plant
	TRINITY_DN902_c0_g1	1.504455192	up	K09422 transcription factor MYB111; K09422 transcription factor MYB, plant
	TRINITY_DN170_c0_g2	-6.925870172	down	K08869 predicted protein; aarF domain-containing kinase
	TRINITY_DN8841_c0_g1	1.445303609	up	K09422 transcription factor MYB41; transcription factor MYB, plant
	TRINITY_DN5067_c3_g1	-1.10759741	down	K09422 transcription factor MYB8; transcription factor MYB, plant
	TRINITY_DN446_c1_g1	-1.583849397	down	K09422 transcription repressor MYB5; transcription factor MYB, plant
	TRINITY_DN81_c2_g2	1.293479038	up	K09422 hypothetical protein; transcription factor MYB, plant
	TRINITY_DN11156_c1_g1	-1.065685029	down	K09422 putative R2R3-Myb transcription factor; transcription factor MYB, plant
	TRINITY_DN11156_c0_g1	-1.30708413	down	K09422 hypothetical protein; transcription factor MYB, plant
	TRINITY_DN8183_c0_g1	-1.578432552	down	K09422 transcription factor MYB63; transcription factor MYB, plant
bHLH	TRINITY_DN2728_c0_g1	-2.177329071	down	K13081 leucoanthocyanidin reductase-like; leucoanthocyanidin reductase
	TRINITY_DN11733_c0_g1	-3.938382332	down	K13081 leucoanthocyanidin reductase-like; leucoanthocyanidin reductase
	TRINITY_DN3592_c2_g1	1.056488843	up	K12126 hypothetical protein; phytochrome-interacting factor 3
	TRINITY_DN5703_c0_g1	-1.584088694	down	K12126 hypothetical protein; phytochrome-interacting factor 3
	TRINITY_DN3769_c0_g1	1.06366355	up	K07953 GTP-binding protein SAR1-like; GTP-binding protein SAR1
	TRINITY_DN5075_c6_g1	1.001935548	up	K13422 hypothetical protein; transcription factor MYC2
	TRINITY_DN10517_c0_g1	-1.145096759	down	K16189 transcription factor PIF4; phytochrome-interacting factor 4

### qRT-PCR validation of differential expression

**Fig. 5:**
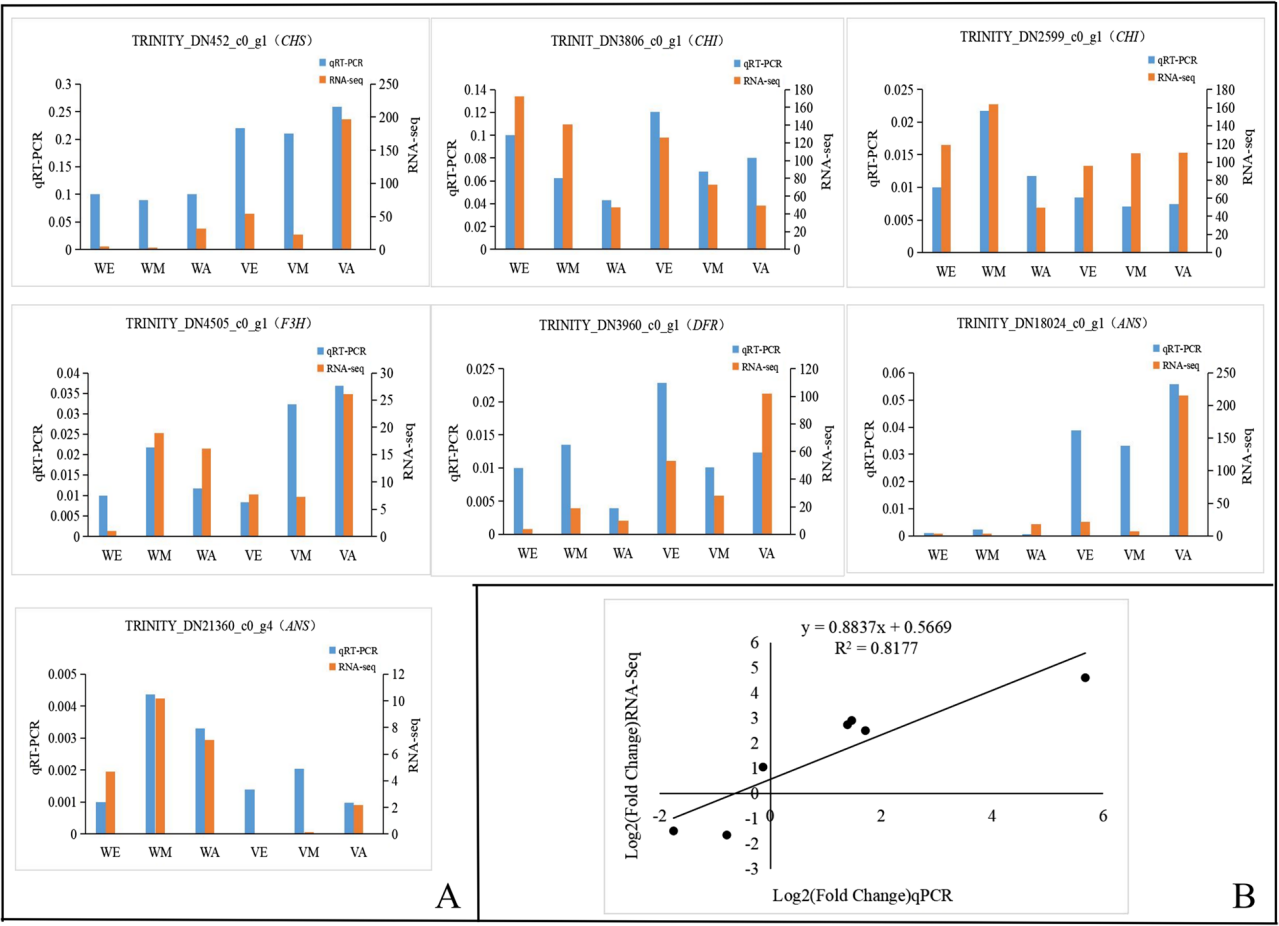
Quantitative RT-PCR analysis of seven candidate genes related to leaf color in *A. pseudosieboldianum.* **A** Quantitative real-time PCR validation of RNA-Seq data of seven genes. **B** The correlation point map from the expression level of FPKM value and qRT-PCR of seven genes

To confirm the unigenes obtained from sequencing and to further validate the reliability of the RNA-seq data, the expression levels of seven genes (including *CHI*, *CHS*, *F3H*, *DFR*, *ANS*) in leaves of six maples were analyzed. The qPCR expression of the seven gene were basically consistent with the transcriptome sequencing results during different stages of the color change in maple leaves (Fig. 5A). The correlation was analyzed from the expression levels of FPKM values and the qRT-PCR of genes related to leaf color in *A. pseudosieboldianum* (Fig. 5B). These results indicate that the transcriptomic analysis was reproducible and reliable, and these candidate DEGs may play important roles in leaf color. Further studies will be conducted to clarify their functions.

## Discussion

### Candidate genes involved in anthocyanin biosynthesis

Given that the *A. pseudosieboldianum* is rich in anthocyanin, we focus on identifying the candidate genes involved in anthocyanin biosynthesis. Flavonoids have various structures and are critical secondary metabolites in many plants. The anthocyanin biosynthesis pathway is an important branch of the flavonoid metabolism pathway and is responsible for anthocyanin production in different plant tissues [22]. Notably, coding enzymes play an important role during this process. However, the overall molecular mechanism of anthocyanin biosynthesis and accumulation in *A. pseudosieboldianum* is not fully understood. In this study, a total of 13 candidate gene-coding enzymes were screened in the flavonoid biosynthesis pathway. CHS is a key enzyme in the biosynthetic pathway of anthocyanins and flavonoids [23]. For example, CHS genes act as the specific key genes regulating flavonoid accumulation in ginkgo leaves [24]. The results of this study showed that one CHS gene showed an increasing trend in the period in WE vs VE, WM vs VM, and WA vs VA. It may be the main reason the color of leaves shows red in spring and summer in the *A. pseudosieboldianum* mutant. This result demonstrates that the biosynthesis of flavonoids is strongly enhanced by CHS expression. Dihydroflavonol 4-reductase (DFR) has been proposed to be an important step in the flavonoid biosynthesis pathway of anthocyanins, and the biosynthesis of anthocyanins in tobacco can be modulated by introducing DFR genes [25]. In this study, one DFR gene was detected and found to be up-regulated in WE vs VE, WM vs VM, and WA vs VA. This suggests that DFR accumulation is an important prerequisite for the accumulation of anthocyanins and flavonoids in the early, middle, and late stages of *A. pseudosieboldianum.* Anthocyanin synthase (ANS) catalyzes the oxidation of colorless cryptochrome anthocyanins to corresponding colored anthocyanins and is a key gene affecting anthocyanin accumulation in plants [26]. A study on the molecular mechanisms of melon peel coloration also found two ANS down-regulated genes involved in the accumulation of anthocyanins. Three ANS genes were found in the present study, and they were more significantly up-regulated in different stages. Therefore, These structural genes influence the formation of maple leaf color, which consistent with other studies [27, 28].

The ANR gene is a key gene involved in proanthocyanidin production in plants [29]. In the present study, two anthocyanidin reductase (ANR) genes (TRINITY_DN11089_c0_g3 and TRINITY_DN12211_c0_g1) were found in all DEGs. TRINITY_DN11089_c0_g3 was found to be up-regulated in WE vs VE and TRINITY_DN12211_c0_g1 was found to be up-regulated in WM vs VM. This promoted the formation of procyanidins and hindered anthocyanin accumulation, which prevented the leaves from appearing red. In short, all structural genes eventually led to the production of a large number of anthocyanins, resulting in the transformation of maple leaf color.

### Candidate TFs involved in anthocyanin biosynthesis

According to previous research performed on plant species, TFs have been proposed to play an important role in regulating the biosynthesis and transport of flavonoids. In particular, the expression of structural genes involved in flavonoid synthesis is largely controlled by basic helix-loop-helix (bHLH), MYB proteins and WD-repeat-containing proteins [30]. Previous studies have concluded that the MYB family plays a major role in regulating sets of genes that are responsible for secondary metabolite biosynthesis pathways in plants. The MYB TF from *Petunia hybrida*, where flavonoid biosynthetic genes are actively expressed, strongly suggests that MYB plays a role in the regulation of flavonoid biosynthesis [31]. MYB111 has been demonstrated to be involved in the regulation of flavonoids biosynthesis in *Arabidopsis* [32]. In the tea cultivar ‘Zijuan’, MYB4, MYB23, MYB26, MYB82, and bHLH74, have been found to be related to anthocyanin synthesis [33]. *Md*MYB114 was confirmed to be significantly positively correlated with anthocyanin content in apple fruit [34]. In our study, 19 MYB were detected that may be related to regulating the expression of leaf color variation. These genes, including MYB111, MYB23, and MYB114 were differentially expressed and may be involved in leaf color regulation in *A. pseudosieboldianum*. The N-terminus of the bHLH TF has a MYB-interacting region (MIR) that interacts with MYB proteins [35]. In maize, this structure plays an important role in color generation and is activated by the interaction between bHLH proteins and R2R3-MYB proteins [36]. Thus, these seven bHLH TFs detected in this study may interact with MYB proteins and consequently affect the synthesis of flavonoids. Also, the MYB-bHLH-WD40 (MBW) ternary complex has been confirmed to play a key role in flavonoid biosynthesis and transport processes because of its regulatory effect on many structural genes [37–39]. MYB, bHLH, and WD40 have been found among the transcriptome of DEGs in the present study and may form an MBW to regulate leaf color. In further studies, the regulation of leaf color variation by these TFs and their interactions will be investigated.

### Candidate genes involved in anthocyanin transport

Anthocyanin synthesis is one of the most thoroughly studied metabolic pathways in biology, but the final molecular mechanism of anthocyanin transport from the cytoplasm to the central vacuole is still unclear [40, 41]. To date, four anthocyanin transport models have been proposed, namely, Glutathione S-transferase (GST), multidrug resistance-associated proteins (MRPs), multidrug and toxic compound excretion associated proteins (MATE), and H + -ATPases, which have also been found to be involved in anthocyanin transport to vacuoles [42]. The most complete possible anthocyanin glycoside transport mechanism is a combination of GST located in the cytoplasm. Transport mechanism research of *Vitis vinifera* has provided evidence suggesting that GST-mediate flavonoid transport is glutathione-dependent [43]. In the present study, six GST genes were significantly expressed and may also be related to flavonoid transport. GST binds to anthocyanin and acts as a transport carrier of anthocyanin, transporting anthocyanin to the vacuolar membrane, and then transporting anthocyanin to the vacuole through MRPs (located on the vacuolar membrane). MATE and H + -ATPases are also important flavonoid transporters. In the present study, one MATE gene and one H + -ATPases gene were found that may be involved in flavonoid biosynthesis regulation.

Although a preliminary understanding of the flavonoid biosynthesis pathway is available, there is still limited information on the transmembrane transport of flavonoids and their accumulation in different compartments. These issues are also the focus of further research.

## Conclusion

Through transcriptome sequencing of *A. pseudosieboldianum*, this study comprehensively analyzed the genes related to the leaf-color mechanism and screened out the candidate genes, differential TFs and transposed-factor candidate genes that determined the flavonoid biosynthesis of leaf color formation. In total, 8,609 DEGs were identified by RNA-seq transcriptome sequencing of *A. pseudosieboldianum* and its mutant. Among these DEGs, 13 structural unigenes encoding seven enzymes that related to the flavonoid biosynthesis pathway were identified, and six GST genes, one MATE gene and one H + -ATPase gene were found to be related to transport of flavonoids. In addition, 19 MYB genes and seven bHLH genes were found to be related to leaf color regulation. These findings provide useful insights into the molecular mechanisms of variants whose leaves turn red in spring and summer and will help researchers obtain more data on pigment synthesis. The findings also present useful information on the deposition of flavonoids and anthocyanins during development, as well as valuable genetic resources for the improvement of leaf colors in the future.

## Methods

### Plant materials

*A. pseudosieboldianum* were cultivated at Yanbian University (129°49 E, 42°92 N), Yanji City, Yanbian Korean Autonomous Prefecture, Jilin Province, China. On May 15, May 30, and June 15, 2021, healthy maple leaves without signs of disease or insect pests were selected from the mutation group and the control group for sampling. The experiment was repeated three times for each sample, with a total of 18 samples (Fig. 1A). All materials were frozen in liquid nitrogen and stored at − 80 °C until use. We declare that the research programme complies with relevant institutional, national and international guidelines and legislation, and we have permission to cultivate *A. pseudosieboldianum*.

### Determination of anthocyanin in leaves

The content of anthocyanin was extracted by spectrophotometer. Approximately 0.2 g of leaf tissue was weighed per sample and placed into a centrifuge tube. Then, 20 ml of 1% hydrochloric acid methanol extract was added into the centrifuge tube. Ultrasonic extraction was performed for 4.5–6.0 h. Centrifugation was followed and the supernatant was taken for further analysis. Spectrophotometer was used to extract anthocyanin in accordance based on the absorbance of the maximum absorption wavelength (535 nm) as measured. Three biological replicates were performed per group.

### cDNA library construction and sequencing

Total RNA was extracted using a mirVana miRNA Isolation Kit (Ambion) in accordance with the manufacturer’s protocol. RNA quantity, purity, and integrity were .

assessed using a Nanodrop-2000 spectrophotometer (GE, Fairfield, CT, USA). Total RNA was measured using an Agilent 2100 Bioanalyzer (Agilent, Santa Clara, CA, USA). After qualified samples were detected, eukaryotic mRNA was enriched by magnetic beads with Oligo (dT) through a-T complementary pairing with the ployA tail of mRNA. Then the mRNA was fragmented into short fragments by fragmentation buffer, and the first-strand cDNA was synthesized using random hexamers reverse transcription with mRNA as template. Then buffer, dNTPs and DNA Polymerase I were added to synthesize two-stranded cDNA. Subsequently, AMPure XP Beads were used to purify double-stranded cDNA. The purified double-stranded cDNA was repaired at the end, a-tails were added, and sequencing joints were connected. Then AMPure XP Beads were used for fragment size selection, and PCR enrichment was performed to obtain the final cDNA library. The libraries were then sequenced on an Illumina sequencing platform (Illumina HiSeq 2500, Illumina, San Diego, CA, USA), and 150 bp paired-end reads were generated.

### De novo assembly and functional annotation

FastQC software and NGS QC Toolkit (v2.3.3) software were used to evaluate the quality of raw data and remove adapter sequences, low-quality, and duplicated reads. After the adaptor and low-quality sequences were removed, using Trinity [44] software obtain clean readings from scratch by assembling transcripts into sequences. BLAST [45] software was used to compare the sequences of unigene with seven public databases (evalue < 0.00001). Including Nr (NCBI non-redundant protein sequences) [46], Swiss-prot (manually annotated and reviewed protein sequence database) [47], GO (gene ontology) [48], KOG (Protein homologous clusters) [49], KEGG (Kyoto Encyclopedia of Genes and Genomes) [50]. KOBAS2.0 [51] was used to obtain KEGG orthology results of unigene in KEGG. After predicting amino acid sequence of unigene, HMMER software was used to compare with FPKM [52] database to obtain annotation information of unigene.

### Differential expression analysis

Bowtie software was used to compare the readings obtained by sequencing with unigene library, and the results obtained by comparison were estimated with RSEM [53] for expression level. The expression abundance of unigene was expressed by FPKM value, which could eliminate the influence of gene length and sequencing quantity difference on the gene expression. Single gene expression level was detected by the method of per thousand base per million reading fragments, and DESeq2 [54] was used for differential expression analysis between sample groups. The screening criteria based on FDR < 0.01 and FC (Fold Change) ≥ 2 were used to judge the significance of gene expression differences and select and obtain the final inter-group DEGs.

### Quantitative real-time PCR (qRT-PCR) analysis

To verify the expression pattern of candidate genes, differentially expressed genes related to anthocyanin synthesis were verified by qRT-PCR. Quantitative analysis was performed using a fluorescence quantitative PCR kit (2 × SYBR® Green premix) and a Gene9600 fluorescence quantitative PCR instrument. The qRT-PCR primers used are listed in Table S9. The gene c110191.graph_c0 was used as a reference gene. The primers used are F: CAACCAGTCTCATCGCAAAT and R: GGCTAACATCCCTTACCAAAT. The following reaction procedure was that initial denaturation at 95 °C for 3 min followed by 39 cycles of 95 °C for 10 s, 58 °C annealing and extension for 30 s. After the reactions, a dissociation curve analysis was conducted to evaluate the primer specificity. The amplification results were analysed using the comparative cycle threshold (Ct) method, which uses the formula 2 − ΔΔCT [55]. The qRT-PCR results were calculated as the means of three replicated treatments. All primers were synthesized by Biotechnology Co. Ltd. Beijing, China.

## Supplementary Information

**Additional file 1: Table S1.** ABC transporters identified in differentially expressed genes. **Table S2.** Glutathione S-transferase (GST) identified in differentially expressed genes. **Table S3.** Multidrug resistance-associated proteins (MRP) identified in differentially expressed genes. **Table S4.** Multidrug and toxic compound excretion associated protein (Mate) identified in differentially expressed genes. **Table S5.** H+-transporting ATPase identified in differentially expressed genes. **Table S6.** MYB identified in differentially expressed genes. **Table S7.** bHLH identified in differentially expressed genes. **Table S8.** WD40 identified in differentially expressed genes. **Table S9.** Designed primers for RT-qPCR.

**Additional file 2: Figure S1.** GO annotation of DEGs (WE vs VE). A. GO enrichment histogram. B. Thumbnail view of directed acyclic graphs (DAGs) of BP, CC, and MF. **Figure S2.** GO annotation of DEGs (WM vs VM). A. GO enrichment histogram. B. Thumbnail view of directed acyclic graphs (DAGs) of BP, CC, and MF. **Figure S3.** KEGG annotation of DEGs (WE vs VE). **Figure S4.** KEGG annotation of DEGs (WM vs VM).

## Data Availability

Raw-reads data were deposited in the NCBI Sequence Read Archive (SRA) with accession number of PRJNA805289.
