# Supplementary material for: De novo transcriptome revealed genes involved in anthocyanin biosynthesis, transport, and regulation in a mutant of Acer pseudosieboldianum
Source: BMC Genomics. 2022 Aug 8;23:567. doi: 10.1186/s12864-022-08815-y (PMC9361605; doi:10.1186/s12864-022-08815-y)
Supplement: Supplementary file 2 — Additional file 2: Figure S1. GO annotation of DEGs (WE vs VE). A. GO enrichment histogram. B. Thumbnail view of directed acyclic graphs (DAGs) of BP, CC, and MF. Figure S2. GO annotation of DEGs (WM vs VM). A. GO enrichment histogram. B. Thumbnail view of directed acyclic graphs (DAGs) of BP, CC, and MF. Figure S3. KEGG annotation of DEGs (WE vs VE). Figure S4. KEGG annotation of DEGs (WM vs VM). [file 12864_2022_8815_MOESM2_ESM.doc]

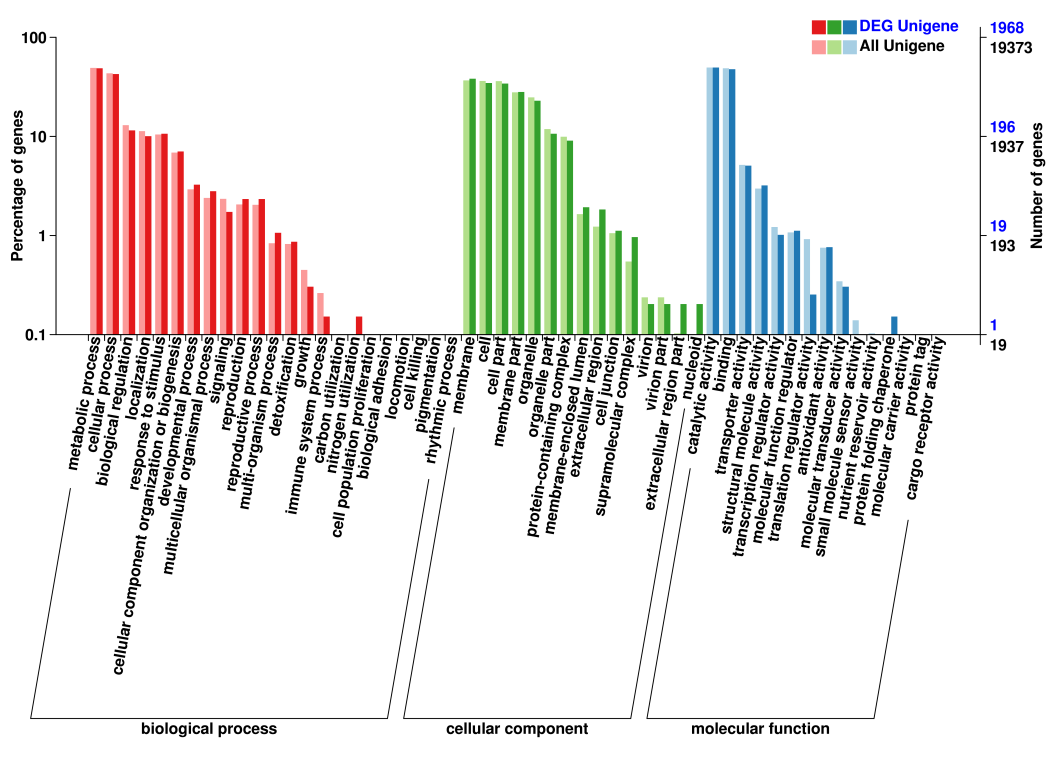


B
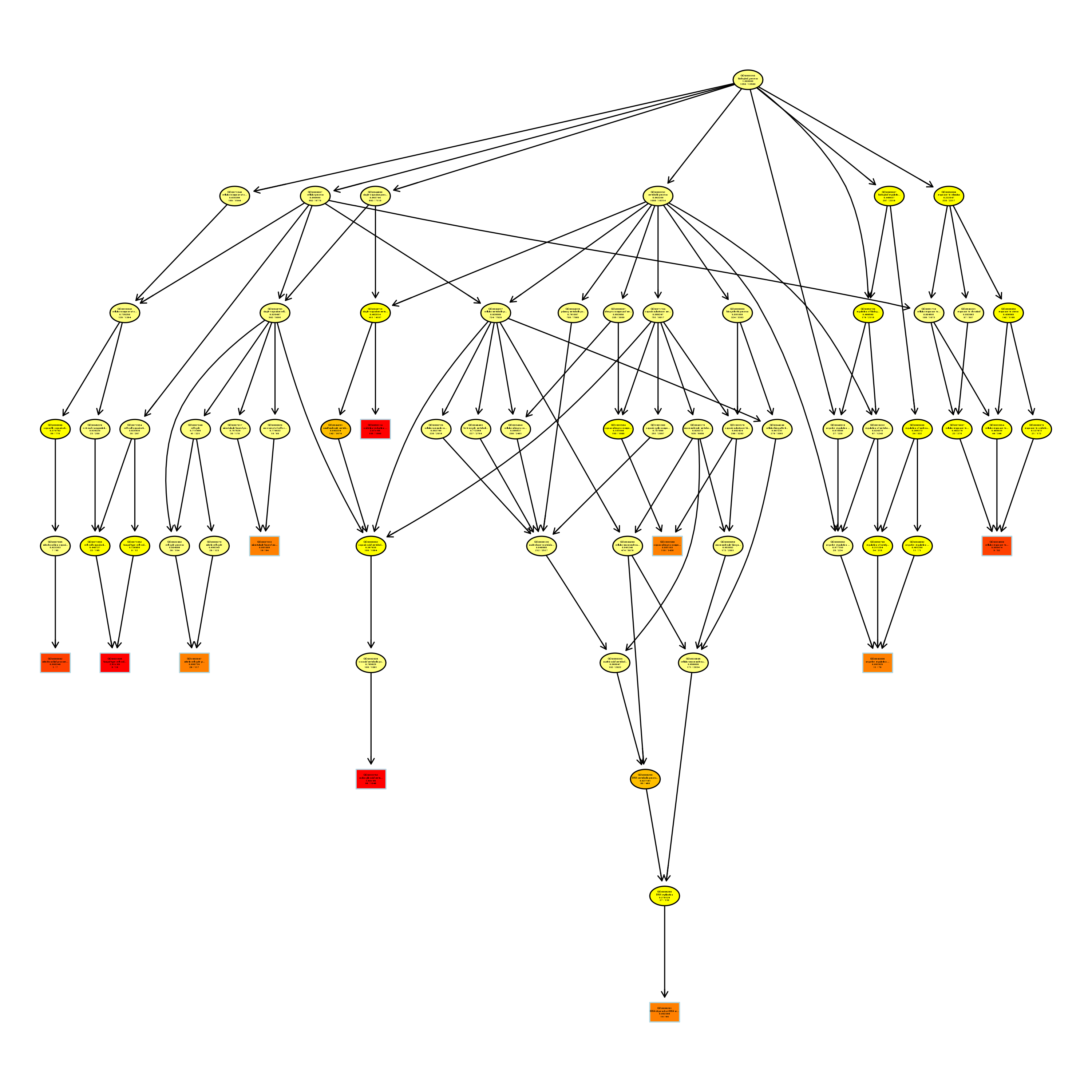

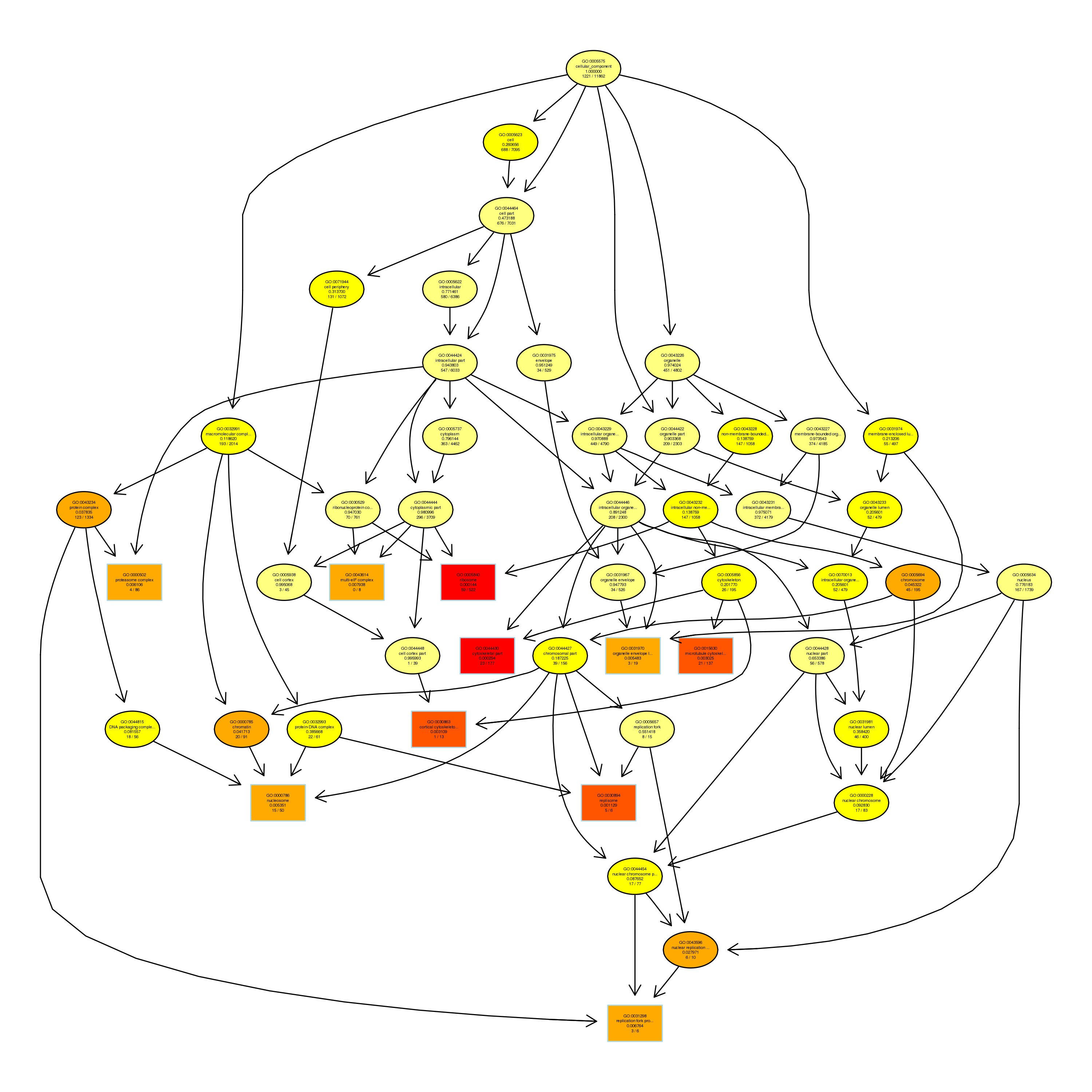

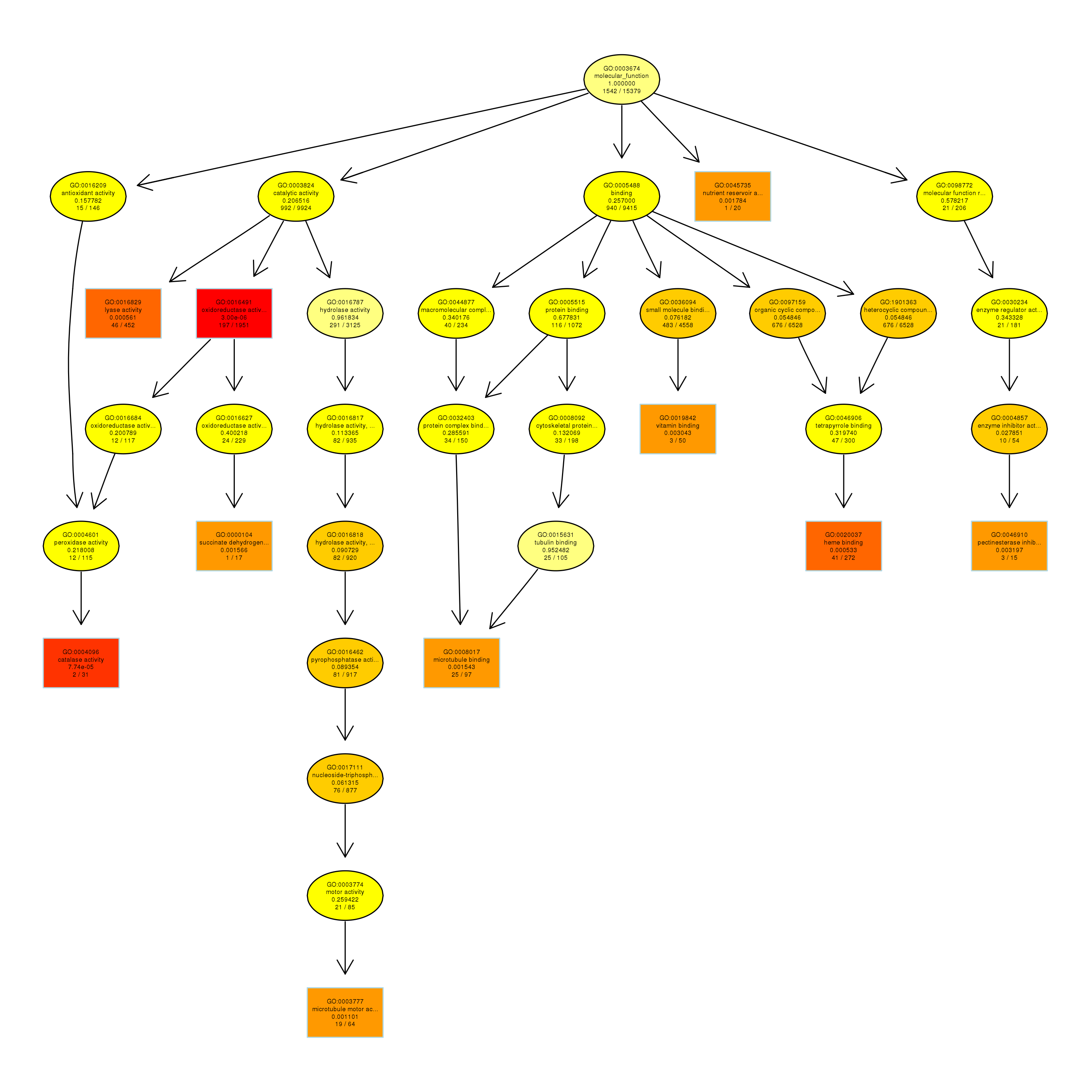


**Figure S1.** GO annotation of DEGs (WE vs VE). **A.** GO enrichment histogram. **B.** Thumbnail view of directed acyclic graphs (DAGs) of BP, CC, and MF.


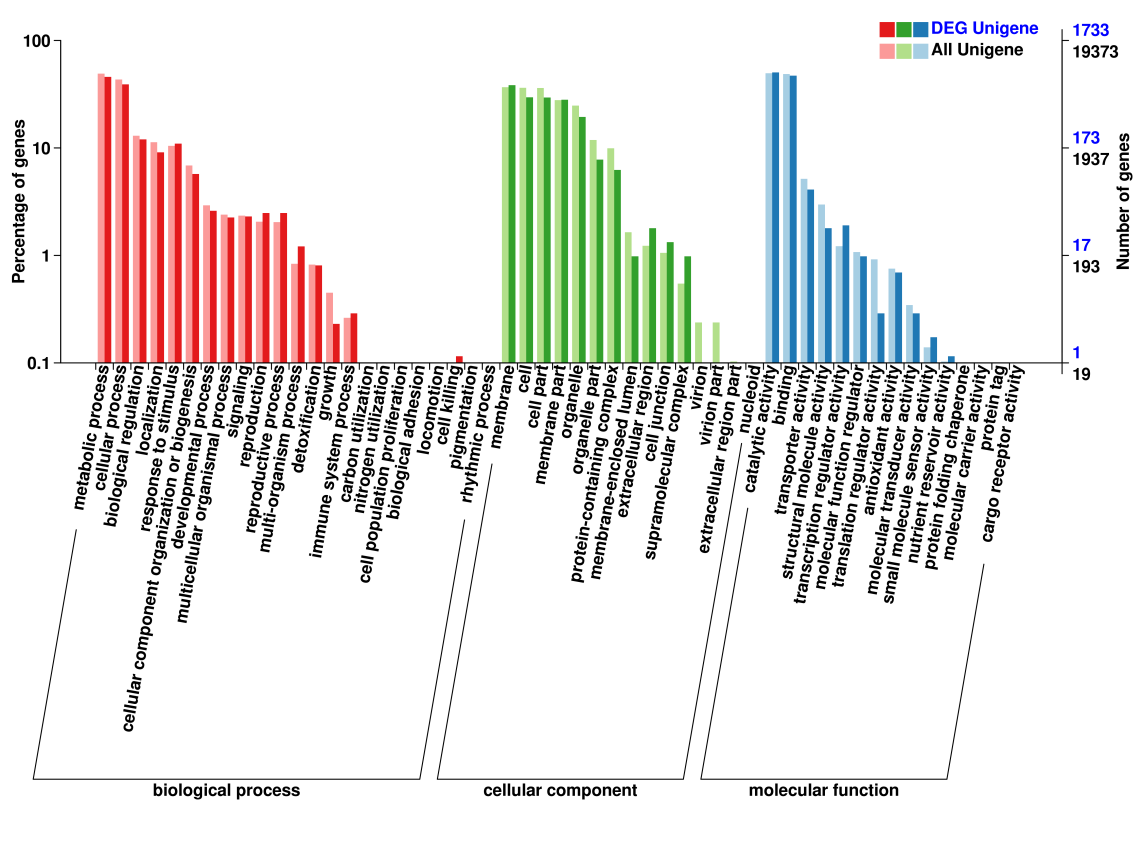


B
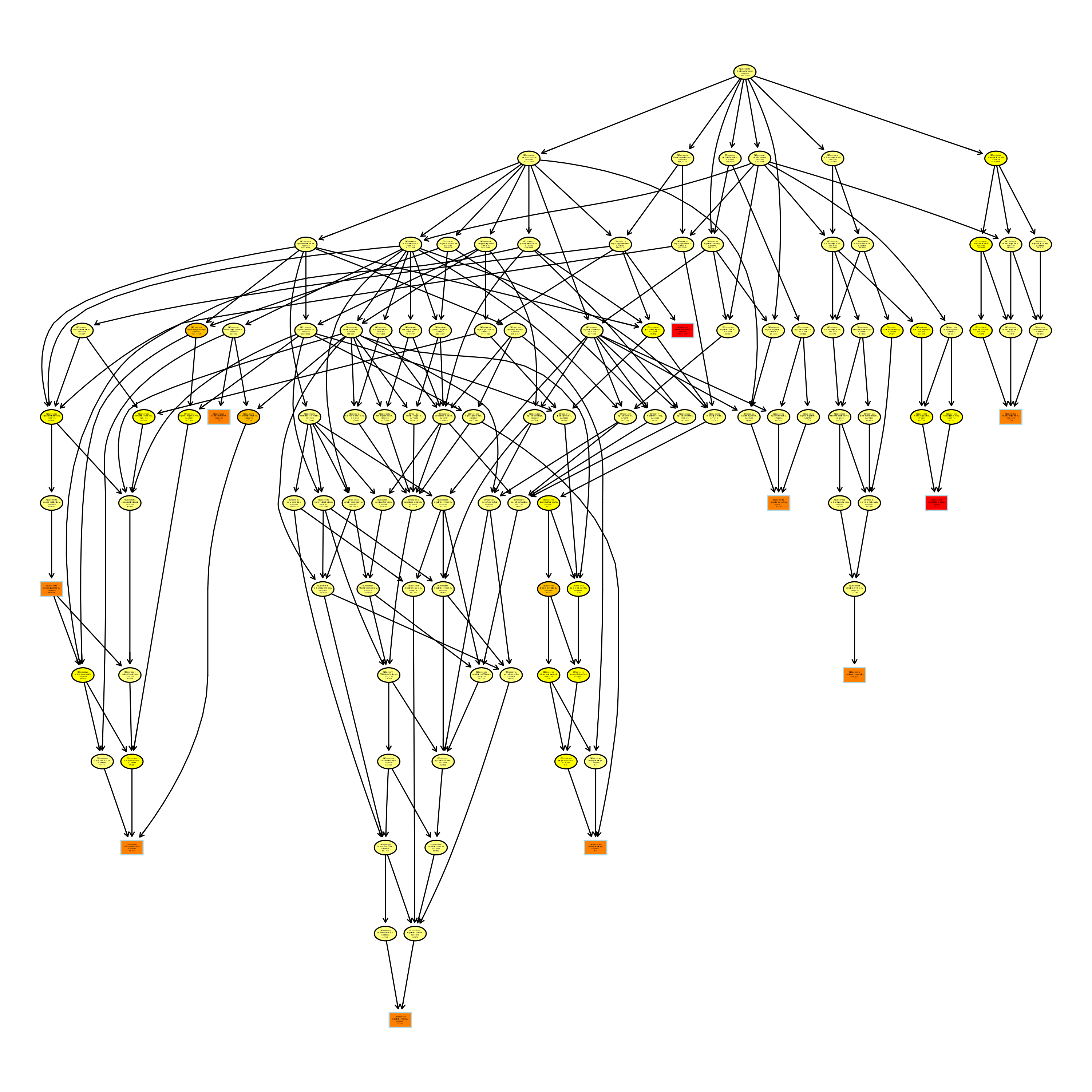

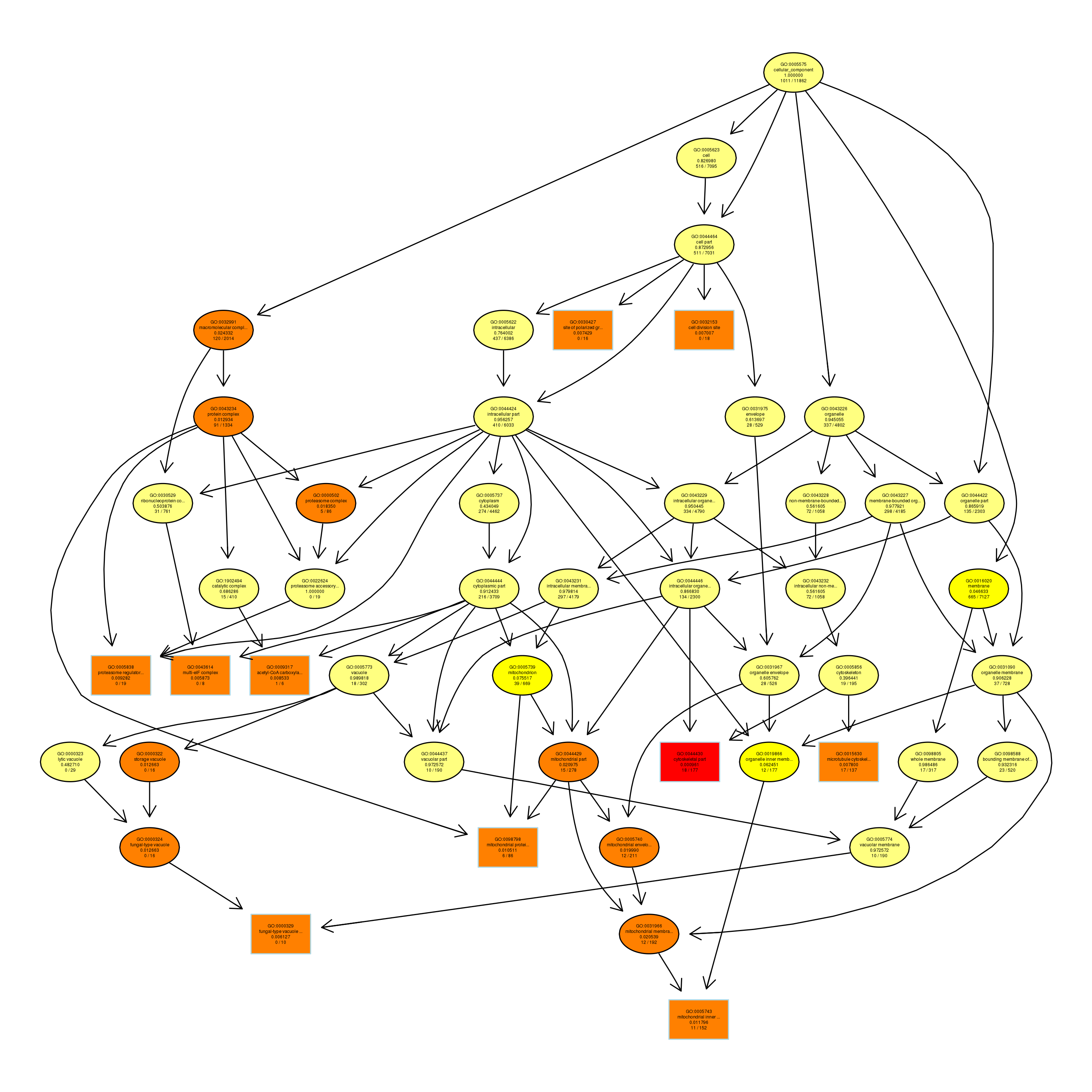

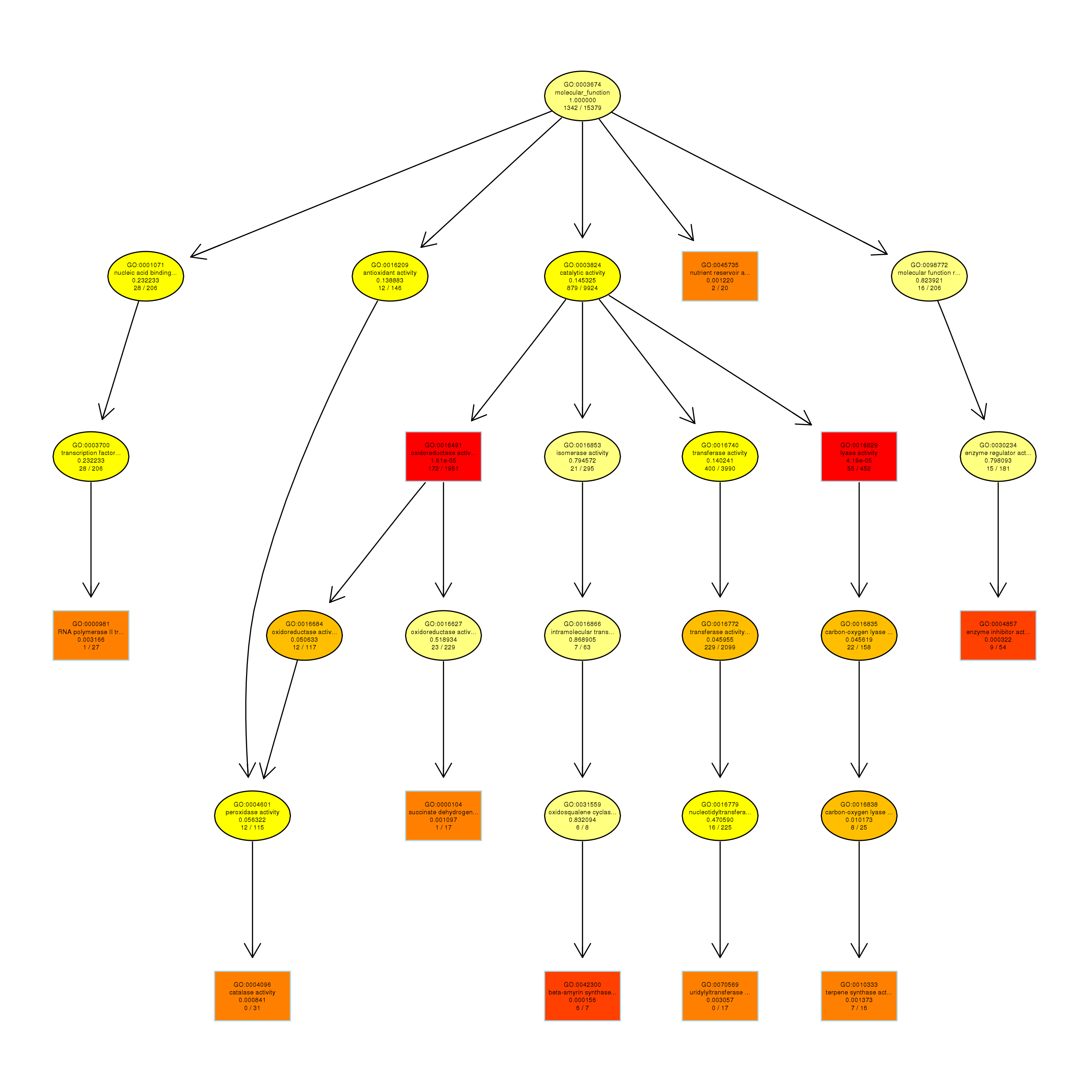


**Figure S2.** GO annotation of DEGs (WM vs VM). **A.** GO enrichment histogram. **B.** Thumbnail view of directed acyclic graphs (DAGs) of BP, CC, and MF.


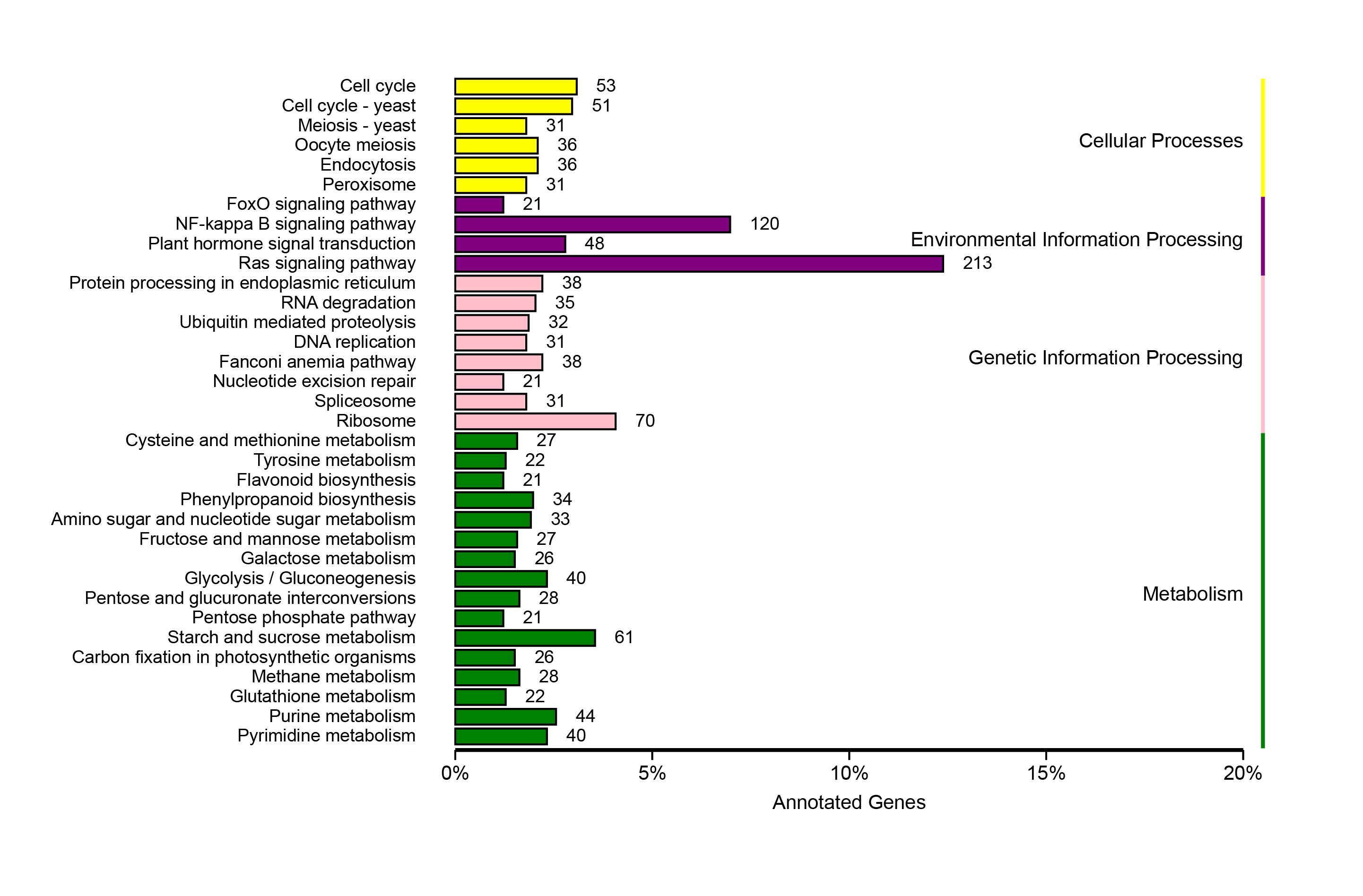


**Figure S3.** KEGG annotation of DEGs (WE vs VE)


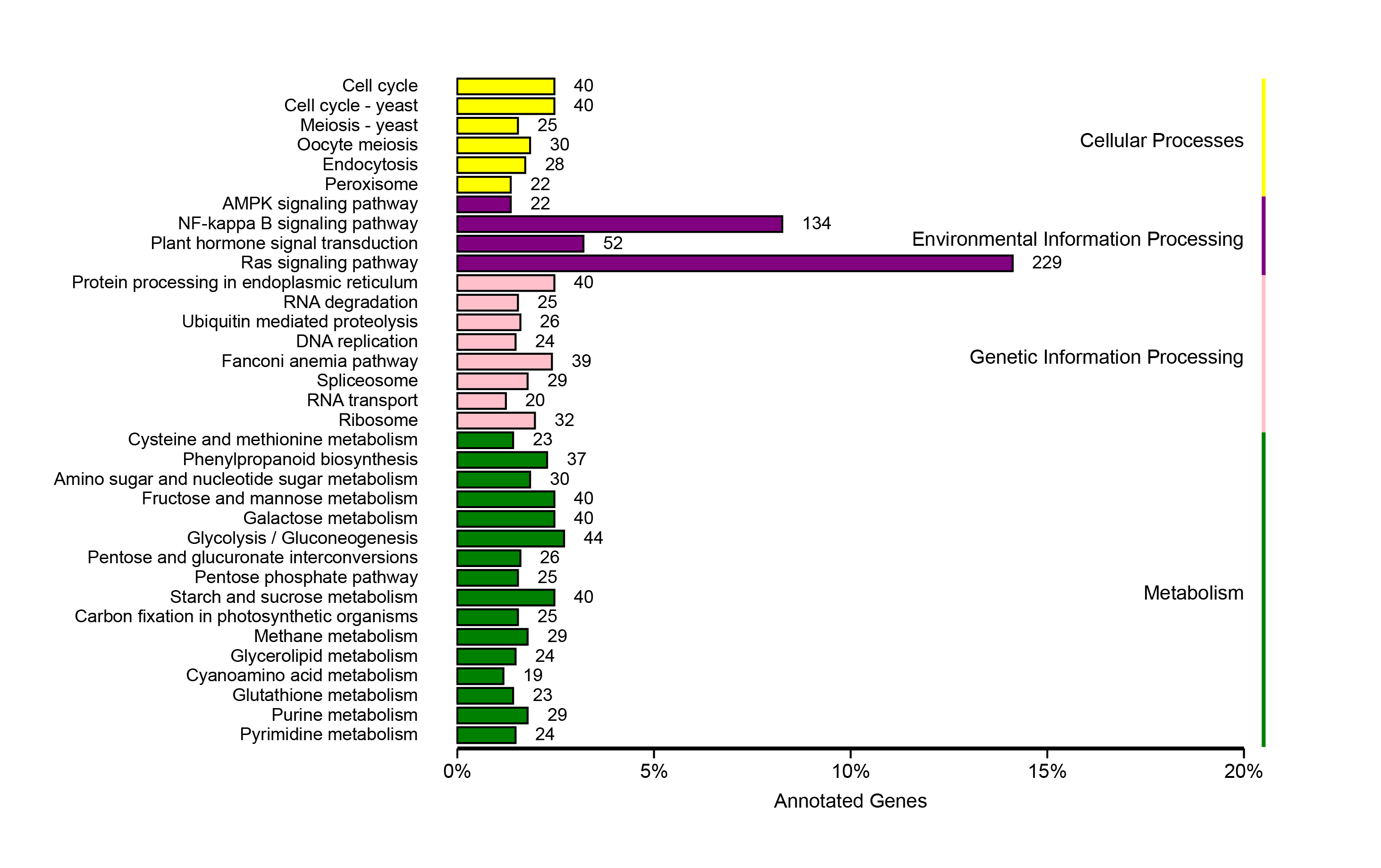


**Figure S4.** KEGG annotation of DEGs (WM vs VM)
